# Percutaneous Patent Foramen Ovale Closure: Stroke and Beyond

**DOI:** 10.2174/011573403X276984240304044109

**Published:** 2024-03-11

**Authors:** Sandeep Randhawa, Jawahar L. Mehta, Gaurav Dhar

**Affiliations:** 1 Division of Cardiology, The University of Arkansas for Medical Sciences, Little Rock, Arkansas, USA

**Keywords:** Patent foramen ovale, cryptogenic stroke, ischemic stroke, patent foramen ovale closure, paradoxical embolism, atrial septal defect, right-to-left shunt

## Abstract

Over 750,000 individuals suffer from stroke annually in the United States, with 87% of these strokes being ischemic in nature. Roughly 40% of ischemic strokes occur in individuals 60 years of age or under. A quarter of all ischemic strokes have no identifiable cause despite extensive workup and are deemed cryptogenic in nature. Patent Foramen Ovales (PFO) has been postulated in stroke causation by either paradoxical embolization or platelet activation in the tunnel of the defect. The incidence of PFO is reported to be 15-25% in the general population but rises to 40% in patients with cryptogenic stroke. While the initial trials evaluating PFO closures were non-revealing, subsequent long-term follow-ups, as well as recent trials evaluating PFO closures in cryptogenic stroke patients 60 years of age or under, demonstrated the superiority of percutaneous closure compared to medical therapy alone, leading to FDA approval of PFO closure devices. In this review, we review the diagnosis of PFO, postulated stroke mechanisms, literature supporting PFO closure, patient selection for percutaneous closure, procedural considerations, and associated procedural complications.

## 1 INTRODUCTION

The annual burden of stroke in the United States (US) is 795,000, with 87% of these strokes being ischemic in nature and approximately 610,000 of these being first-time strokes [1]. The direct medical cost for the diagnosis and treatment of strokes in the US was estimated at $33.4 billion between 2017-2018, with the direct and indirect costs amounting to $52.8 billion [1]. These costs include not only direct medical care, including hospital stays, emergency visits, office-based visits, and home health care, but also long-term disability and loss of income. A cryptogenic stroke is an ischemic stroke where no probable cause is identified after appropriate diagnostic evaluation. These account for roughly 25-40% of all ischemic strokes, with a UK-based population study reporting 32% incidence amongst the 2555 patients studied with a first-time transient ischemic attack or ischemic stroke [2, 3]. While the overall prevalence of a Patent Foramen Ovale (PFO) in the general population has been described as 25-30%, the prevalence was significantly higher at 40% when evaluating patients with cryptogenic stroke [4-6]. The association between PFO and cryptogenic stroke is especially more prominent when evaluating patients younger than 55 years of age [7]. We provide an update to prior review on this subject by Collado *et al.,* including the 5-year follow-up data from REDUCE trial [8, 9].

## 2 METHODOLOGY AND LITERATURE SEARCH

The present study was prepared in accordance with the Preferred Reporting Items for Systematic Reviews and Meta-Analyses (PRISMA) statement [10]. The primary literature search for randomized studies evaluating the role of PFO closure in cryptogenic stroke was conducted in SR and GD. We searched the Cochrane Library and Pubmed for all articles published between 1995 and January 2023 using the search terms “Patent Foramen Ovale,” “Cryptogenic Stroke,” “Atrial septal defect,” and “Percutaneous closure.” A total of 155 studies were reviewed, and 6 randomized trials, with 2 of them having long-term follow-up addressed percutaneous PFO in cryptogenic stroke were included.

## 3 PFO DIAGNOSIS AND CLASSIFICATION

A PFO is a potential space in the atrial septum when there is an incomplete postnatal fusion of septum primum and secundum, leading to an intermittent or continuous right-to-left shunt. It is diagnosed on transthoracic echocardiogram (TTE), transesophageal echocardiogram (TEE) or intracardiac echocardiogram (ICE) by using color flow doppler, spectral doppler or agitated saline contrast to visualize shunting across the septum. A TTE is usually the initial diagnostic modality of choice, and micro-bubbles from agitated saline, along with provocative maneuvers to, increase right atrial pressures and increase the diagnostic accuracy of the test. The visualization of microbubbles in the left atrium within the first 3-6 cardiac cycles after opacification of the right atrium is considered diagnostic of an intracardiac shunt such as a PFO, whereas opacification after 6 cardiac cycles is usually indicative of an intrapulmonary shunt [11].

Once identified, a complete evaluation of PFO requires defining the anatomy, including the size/ height of PFO, the length of the tunnel, the right and left atrial rims, as well as the presence of associated atrial abnormalities [12]. Grading of shunting is often performed using a scale described by Rana *et al.* by counting the number of microbubbles appearing in the left atrium. Grade 1 is <5 bubbles, grade 2 is 5 to 25 bubbles, grade 3 is >25 bubbles, and grade 4 is the opacification of the chamber [13].

## 4 POTENTIAL STROKE MECHANISM IN PFO

Several potential mechanisms have been hypothesized to explain stroke in PFO patients. The initial hypothesis suggested paradoxical embolization of a venous thrombus through the PFO (right to left shunt), leading to arterial embolization of the thrombus to cerebral circulation, which has been widely criticized [14, 15]. Lamy *et al.* reported a series of 581 patients with cryptogenic stroke with or without associated PFO where the incidence of deep vein thrombosis was no more frequent in patients with an associated PFO [16]. The other widely accepted hypothesis supports the notion of *in-situ* thrombus formation within the PFO tunnel [17-19]. Atrial tachy-arrhythmias, including atrial fibrillation (AF) and atrial flutter, have also been postulated as possible mechanisms since their incidence was significantly higher in cryptogenic stroke patients with a PFO and atrial septal aneurysm (ASA) [20-22]. An ASA is a deformity of the atrial septum associated with increased mobility and is defined by a 10 mm excursion from the septal plane into the right or left atrium or by a combined 15 mm excursion [12].

Amongst patients with cryptogenic stroke, several high-risk anatomical atrial and PFO factors have been clearly established. A PFO tunnel size (height) ≥ 2 mm, tunnel length ≥ 10 mm, ASA, hypermobile interatrial septum, prominent Eustachian valve and presence of Chiari network are associated with an increased risk of stroke [23, 24]. A greater right-to-left shunting driven by the pressure gradient and not the actual shunt size has been postulated to be associated with increased risk of stroke, although prior studies have revealed conflicting data [19, 24-28].

## 5 HISTORY OF PFO CLOSURE

The first description of a PFO being associated with stroke was provided by Julius Cohenheim in 1877 when he reported a large PFO in a 35-year-old female who passed away from a large cerebral infarct with extensive peripheral venous thrombosis. Subsequently, many case reports in the early 1900s described the incidental finding of PFO in patients with acute stroke with no other obvious cause. The first operative surgical closure of atrial septal defect (ASD) in literature was performed by Blakemore in 1939 and described by Murray in 1948 [28]. The first percutaneous closure of an ASD using an umbrella device was described by Kings and Mills in 1974, and the initial case series of transcatheter PFO closure in human subjects with presumed paradoxical embolus was described by Bridges *et al* [29, 30]. Schuchlenz *et al.* reported an observational case series describing a decreased incidence of recurrent stroke in cryptogenic stroke patients undergoing PFO closure compared to medical therapy with antiplatelet or anticoagulation [31].

Although the use of percutaneous PFO closure was restricted to Humanitarian Device Exemption (HDE) prior to 2006, multiple observational studies at the time demonstrated the benefit of PFO closure over medical therapy. Given the increasing data favoring PFO closure as well as >4000 annual percutaneous closure procedures being performed, the Food and Drug Administration (FDA) revoked HDE in late 2006. This was done to increase patient recruitment into randomized trials, although off-label use for PFO-closure was still allowed. Multiple randomized controlled trials involving devices from different manufacturers have since been published, as summarized in Table **1**, including CLOSURE 1 (2012), PC Trial (2013), RESPECT Trial (2013), CLOSE Trial (2017), REDUCE Trial (2017) and DEFENSE-PFO Trial (2018) [32-37].

CLOSURE 1 trial was a multi-centered, open-labelled, randomized trial comparing STARFlex Septal closure system *vs.* medical therapy (aspirin, warfarin or both) in patients aged 18-60 years of age who presented with cryptogenic stroke or Transient Ischemic Attack (TIA) with composite primary endpoint of stroke or TIA at 2-years [32]. This trial was primarily limited by the low rate of successful device implantation as well as the lack of a standardized description of cryptogenic stroke at the time of the study [32]. PC Trial (2013), funded by St. Jude Medical, was a multi-center randomized, superiority trial that included patients aged 60 years or less with documented PFO on TEE and cryptogenic stroke or peripheral thromboembolism comparing percutaneous closure with Amplatzer PFO occluder *vs.* medical therapy (antithrombotic therapy with acetylsalicylic acid dose of 100 to 325 mg daily for at least 5-6 months, ticlopidine 250 to 500 mg daily, clopidogrel 75 to 150 mg, or oral anticoagulation at discretion of treating physician). Although a negative trial, it had several limitations, including a lack of standardized treatment in the medical therapy group, poor retention and potential under-reporting of events in the device group.

RESPECT Trial was the first multi-center, randomized, event-driven trial with prolonged follow-up at 2.6 years and 5.9 years, respectively, that evaluated 980 patients randomized to device group or medical therapy group (one or more antiplatelets in 75% of patients and anticoagulation in 25% of patients) with a primary composite endpoint of recurrent nonfatal ischemic stroke, fatal ischemic stroke, or early death after randomization [32-38]. While this was a landmark trial, being the first trial with decreased events in the device arm, the medical therapy arm experienced a higher drop-out rate, and no significant difference was observed in intention-to-treat analysis initially.

CLOSE Trial (2017) evaluated 663 patients aged 16-60 years with stroke attributed to PFO with an associated atrial septal aneurysm or large inter-atrial shunt (high-risk PFO) treated with device therapy (any approved PFO device) *vs.* antiplatelets (aspirin or clopidogrel or aspirin with dipyridamole-ER) *vs.* anticoagulation (direct oral anticoagulants or warfarin) with a mean follow-up of 5.3 years and the primary endpoint of recurrent fatal or non-fatal strokes [35]. Although device closure was superior in this trial, enrollment was terminated prematurely (originally intended enrollment was 900 patients) due to a lack of funding and a lack of prolonged monitoring for occult AF.

REDUCE Trial was the second randomized trial to report a short-term as well as a mean long-term follow-up of 5 years and included a total of 664 patients aged 18-59 years of age diagnosed with PFO who suffered a cryptogenic stroke within 180 days before randomization [9, 36]. Patients were randomized in a 2:1 ratio to undergo PFO closure (Helex Septal Occluder or Cardioform Septal Occluder) plus antiplatelet therapy (aspirin, clopidogrel, or a combination or aspirin and dipyridamole) *vs.* antiplatelet therapy alone with a primary endpoint of recurrent clinical ischemic strokes and a co-primary endpoint of new-brain infarcts (clinical ischemic strokes as well as imaging evidence of silent brain infarctions). Both recurrent ischemic strokes and new brain infarcts were significantly lower in the device therapy arm, but results were partly limited due to patients in the medical therapy arm undergoing PFO closure outside trial (n=14).

DEFENCE-PFO Trial was a randomized, open-labelled, superiority trial conducted at 2 sites in South Korea from June 2011 to October 2017 that included 120 patients with cryptogenic stroke and high-risk PFO randomized to device therapy (Amplatzer PFO occluder) or medical therapy arm (Aspirin, or Aspirin and clopidogrel, or aspirin and cilostazol, or warfarin) with a composite primary endpoint of stroke, vascular death or Thrombolysis in Myocardial Infarction defined major bleeding during a 2-year follow-up. Device therapy was found to be superior, with 0 events reported in the device arm and 12.9% in the control arm, driven primarily by ischemic stroke. The results, although impressive, are limited by underpowered studies due to early termination (for patient safety) as well as the publication of consecutive clinical trials favoring PFO closure. Also, the generalizability of the study may be limited due to the patient population and enrollment being limited to 2 centers.

While most trials prior to 2013 showed no difference between percutaneous closure and medical therapy, all randomized trials published in 2017 and onwards (including 5-year follow-up data from the REDUCE Trial and RESPECT trial, summarized in Table **2**) demonstrated the superiority of percutaneous closure compared to medical therapy alone [9, 38]. This led to the FDA approval of percutaneous PFO closure devices, including the Amplatzer PFO occluder on October 28, 2016, and the Gore Cardioform septal occluder on March 30, 2018.

## 6 PATIENT SELECTION

FDA mandates independent evaluation of patients by a cardiologist as well as a neurologist (ideally stroke neurologist) and an informed risk-benefit discussion with a patient before undergoing PFO occluder implantation. The Heart-Brain team approach ensures appropriate patient selection while avoiding the risk of potential harm from inappropriate device implantation in unsuitable candidates. A careful and thorough evaluation to rule out other causes of stroke, including atherosclerotic disease, cardiac source of embolism including AF, aortic dissection, and hypercoagulable states, should be performed. Extended cardiac monitoring to rule out underlying occult AF is especially important given multiple prior trials, including CRYSTAL-AF, EMBRACE and a 3-year follow-up of CRYSTAL-AF demonstrating increasing detection of AF with longer monitoring periods, from 1.9% at 24 hours, 12.4% at 12 months and 30% at 3 years when evaluating patients older than 40 years of age with cryptogenic stroke [39-41]. The 30-day AF rate difference was particularly remarkable when evaluating patients 40 years and older (8.9%) *vs.* patients 50 years and older (16.1%) [40, 41].

The decision to close a PFO is based on patient risk stratification, which is commonly determined utilizing scoring systems. The risk of paradoxical embolism (RoPE) score proposed by Kent *et al.* remains the most widely used scoring system, as demonstrated in Table **3** [42]. Other scoring systems taking into account the PFO anatomy are validated but not as commonly used (20).According to the 2019 Society for Cardiovascular Angiography and Interventions guidelines for the management of PFO, percutaneous closure is indicated in patients with PFO-associated cryptogenic stroke with greatest benefit in patients 18-60 years of age and a RoPE score ≥7 [43]. This was demonstrated by Morais *et al.* in a single-centered study involving 403 patients where a RoPE ≤6 was an independent predictor of higher mortality and recurrent ischemic stroke [44]. Elgendy *et al.* have since proposed the PFO-associated stroke causal likelihood (PASCAL) classification system, which integrates the RoPE score along with imaging findings (straddling thrombus, ASA and PFO shunt size) to classify PFO as being pathogenically being associated with stroke although it has not been integrated into guidelines [45].

## 7 PROCEDURAL CONSIDERATIONS

The Society of Cardiovascular Angiography and Interventions published an expert consensus statement on operator and institutional requirements for percutaneous PFO closure with operators requiring >50 lifetime structural or congenital catheter-based interventions, which includes a minimum of 25 procedures involving atrial septal interventions or 12 specific to PFO closure [43]. Most patients undergoing a PFO closure are pretreated with aspirin (81-325 mg) and clopidogrel (75 mg) on the day of the procedure prior to procedure initiation, given the protocol used in device trials, although this is often left to the discretion of the operating physician [33, 34]. The patients should receive a single dose of intravenous antibiotic within 1 hour of the procedure, typically a weight-based dose of Cefazolin. The procedure is most often performed with ultrasound guided right femoral vein access and anti-coagulation achieved using intravenous unfractionated heparin to target an activated clotting time of over 250 seconds. Alternate vein access has been described when inferior vena cava obstruction has precluded the use of femoral vein access [46-49]. Venous access also depends on procedural imaging guidance. Although most initial procedures were performed under TEE guidance, the increasing use of ICE for peri-procedural imaging, as first described by Hijazi *et al.* would mean the need for a second venous access, typically with ipsilateral or contralateral femoral vein [50]. The venous sheath size depends on the size of the ICE catheter as well as the PFO size and device deployed with a phased-array ICE probe usually being an 8 or a 10-French catheter.

Among the two commercially available PFO closure devices approved by the FDA, the Gore Cardioform Septal Occluder (W.L. Gore and Associates, Inc, Newark, DE) is available in 3 sizes (occluder size 20 mm, 25 mm, and 30 mm) as seen in Fig. (**1**) with catheter size being 10-French and recommended sheath size being 12-French. The Amplatzer PFO Occluder (Abbott Structural, Santa Clara, CA) is available in 4 sizes (Right atrial disc diameter 18 mm, 25 mm, 30 mm, and 35 mm) as seen in Fig. (**2**), with catheter size being 8 or 9-French and the recommended sheath size being 10 to 11-French. The 35-mm device is usually reserved for severely mobile ASA, lipomatous septum secundum and large tunnel diameters (> 4 mm).

Once venous access is established, imaging (ICE *vs.* TEE) is used to re-confirm the position and size of PFO. A 6-Fr multipurpose angiographic diagnostic catheter is then advanced in the left anterior oblique cranial view over a 0.035” J-tipped guidewire, which is used to cross the PFO before being exchanged for a stiff wire to assist delivery of the device and delivery sheath through the PFO into the left atrium. Rarely, balloon sizing may be performed prior to the introduction of a device to accurately assess PFO size. Once the device delivery sheath is introduced into the left atrium, the left disc is deployed, followed by a pullback to the interatrial septum and subsequent right disc deployment in the right atrium. After deployment, confirmation of the adequate position with imaging is performed to visualize the capture of all rims as well as exclude impingement of valvular apparatus prior to device release and delivery sheath removal. Imaging is performed again to confirm device stability, closure of interatrial communication, assessment of mitral and tricuspid valve, and left ventricular function, as well as rule out pericardial effusion. At this time, venous sheaths are removed, and hemostasis is achieved, typically using a Z-stitch in our practice.

Follow-up imaging with TTE is performed at 1-day post-implantation to confirm stable device position and rule out residual shunt. A 6-month follow-up TEE using bubble study is performed to rule out any residual shunt as most devices endothelialize over this time. The recommended duration of dual antiplatelet therapy is at least 1 month post-procedure, with pre-procedural antibiotic prophylaxis recommended for any procedure for up to 6 months after device implantation unless residual shunt is demonstrated [51, 52]. There is no consensus at present regarding either the duration of dual antiplatelet therapy beyond 1 month or the need and/or duration for long-term antiplatelet therapy.

## 8 COMPLICATIONS

With increasing operator experience as well as improving device design and delivery, complications with PFO closure are now becoming rare. These complications can be divided into peri-procedural complications and long-term complications. Immediate peri-procedural complications related to access site include hematoma formation, retroperitoneal hemorrhage, pseudoaneurysm and vascular injury requiring surgical intervention, although the incidence has decreased over time. Potential cardiac complications during device deployment include pericardial effusion, transient ST elevations, device dislodgement, air embolism, intracardiac thrombus formation, pacemaker lead entrapment and cardiac perforation, although the reported overall incidence is less than 0.5% [9, 32-38, 53]. Although the bleeding risk is elevated in the perioperative period in patients undergoing device closure, this was offset by the increased bleeding risk of long-term antiplatelet/ antithrombotic therapy in the medical treatment arm with no trials demonstrating an increased bleeding risk in patients undergoing PFO closure [9, 32-38]. Long-term complications include device thrombosis, pulmonary embolism, nickel allergy, residual shunt, and atrial fibrillation. Nickel allergy can present with post-procedural chest pain, palpitations or worsening migraine headaches and was described previously in patients undergoing PFO or ASD closure, which improved with resuming clopidogrel or device explanation [54, 55]. However, no cases of nickel allergy were found in the 6 major randomized controlled trials evaluating PFO closure for cryptogenic stroke [32-37]. Residual shunt is diagnosed when a follow-up echocardiogram, usually performed at 6 months, demonstrates a flow of bubbles across the interatrial septum with most trials quantifying ≥ 10 bubbles as persistent shunt (≥ 6 bubbles was quantified as persistent shunt in PC Trial) [32-37]. While the initial CLOSURE trial demonstrated a 13.9% incidence of residual shunt in 2012, the DEFENSE-PFO trial reported 100% closure rates with 0% residual shunt in 2018 [32, 37]. This difference may be ascribed to increasing operator experience and improved device design. However, Marchese *et al.* described larger PFO size and the presence of ASA as being independent predictors of the residual shunt in an observational study of 127 patients [56]. Atrial fibrillation is the most commonly reported complication following PFO closure, with a recent meta-analysis reporting an incidence of 3.7% per 100 patient-years in patients undergoing percutaneous closure compared to 0.1 per 100 patient-years in medically treated patients [57]. Interestingly, most of these episodes occurred within 45 days of PFO closure and were transient in a majority of these patients, with increased incidence with advancing patient age [32, 35, 36]. On the contrary, there was no statistical difference in the incidence of atrial fibrillation in patients undergoing PFO closure in the PC trial and RESPECT trial [33, 34]. This mechanism for atrial fibrillation may be explained by local irritation of atrial tissue by the device, although the risk may be different for different devices, with the lowest risk demonstrated with Amplatzer PFO Occluder. Also, the presence of PFO, as well as ASA size and location, are independent risk factors for the occurrence of atrial fibrillation [39, 58]. Management of such patients can be challenging as there is no current consensus on the duration of anticoagulation and long-term monitoring once anticoagulation is stopped [58-61].

## CONCLUSION

From the advent in 1992 to advancements in 2023, percutaneous transcatheter PFO closure has transformed the care of PFO-associated cryptogenic stroke patients, demonstrating superiority to medical therapy. This must be interpreted cautiously, nevertheless, as high-risk patients may have been treated outside trial as well as short-term focus of most reported trials with the average follow-up being only 3.8 years. Furthermore, adherence to assigned therapy following randomization is generally strictly enforced in clinical trials with close follow-ups, which may not be achieved in routine clinical practice. Although PFO closure has also been evaluated in the treatment of migraine with aura (including the MIST trial, MIST II trial, and PREMIUM trial), decompression illness, platypnea-orthodeoxia syndrome and patients with systemic embolism without PFO-associated strokes, current guidelines only recommend PFO closure consideration in patients with platypnea-orthodeoxia syndrome and systemic embolism without PFO associated strokes. A careful patient selection amongst PFO-associated cryptogenic stroke patients with emphasis on RoPE score continues to be crucial as higher RoPE scores ≥7 have consistently been associated with higher attributable stroke risk and lower recurrent strokes after undergoing closure. Performing early closure in this subset of patients offers the promise of longer protection from PFO-associated strokes and associated morbidity. At the same time, no device at present ensures complete and permanent stroke protection, and long-term follow-up is essential given the relatively young age of technology. With improvements in delivery techniques, periprocedural imaging, device technology, and operator experience, effective closure rates have continued to improve with a decreased need for a second operator for TEE and reduced procedural complications. However, the optimal anti-platelet therapy after device implantation, as well as the duration of dual anti-platelet therapy followed by a single anti-platelet regimen, continues to be an area of uncertainty. Moreover, there is a lack of consensus on the management of atrial fibrillation post PFO closure and further research regarding the duration of therapy as well as arrhythmia monitoring is needed. Nonetheless, the future for PFO closure appears promising, with two new devices undergoing clinical trials at present, including the Occlutech Flex II PFO Occluder as well as suture-based NobleStitch EL.

## Figures and Tables

**Fig. (1):**
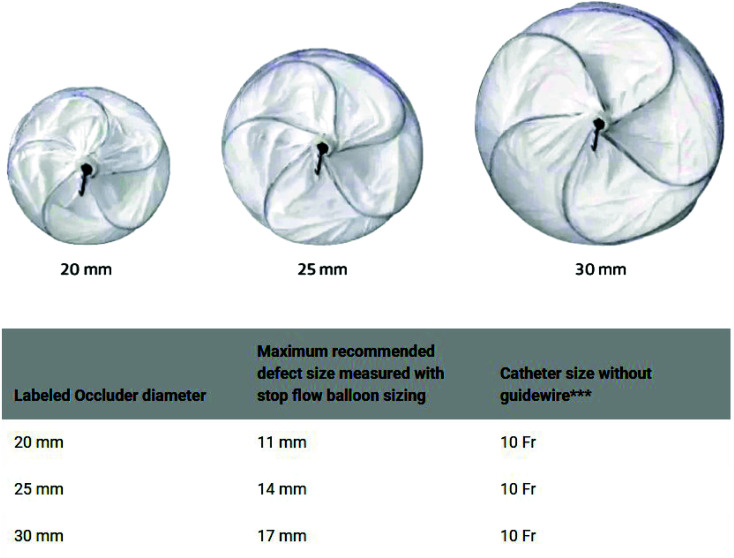
Available sizes, defect size and recommended catheter size for Gore Cardioform Septal Occluder (W.L. Gore and Associates, Inc, Newark, DE).

**Fig. (2):**
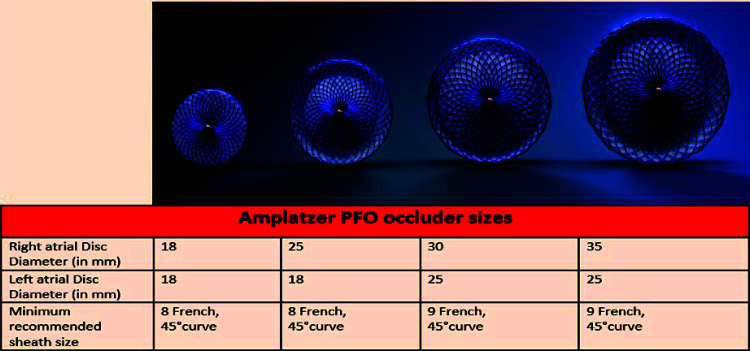
Available sizes and recommended catheter size for Amplatzer PFO Occluder (Abbott Structural, Santa Clara, CA).

**Table 1:** Overview of percutaneous patent foramen ovale closure randomized trials.

**Study, Year and Size**	**Closure Device**	**Mean Follow-up**	**Primary Endpoint**	**Results**	**Conclusion**
CLOSURE1 (2012) [31] n = 909	STARFlex Septal Closure System *vs.* Aspirin, Warfarin* or both	2 years	Composite of death from any cause (0-30 days), neurological death (≥31 days) and Stroke or TIA at 2-year follow-up	5.5% in device arm 6.8% in control arm HR 0.78 95% CI 0.45 to 1.35 *p* = 0.37	Device closure is not superior to medical therapy alone
PC Trial (2013) [32] n = 414	Amplatzer PFO Occluder *vs.* antithrombotic or anticoagulation	4.1 years	Composite of death, nonfatal strokes, TIA, or peripheral embolism	3.4% in device arm 5.2% in control arm HR 0.63 95% CI 0.24 to 0.62 *p* = 0.34	Device closure is not superior to medical therapy alone
RESPECT (2013) [33] n = 980	Amplatzer PFO Occluder *vs.* Aspirin, clopidogrel, warfarin* or aspirin plus dipyridamole	2.6 years	Composite of recurrent nonfatal ischemic stroke, fatal ischemic stroke, or early death after randomization	Intention to treat Event rate 0.66% per 100 patient years in device arm *vs.* 1.38% per 100 patient years in control arm HR 0.49 95% CI 0.22 to 1.11 *p* = 0.08 As treated. Event rate 0.46% per 100 patient years in device arm *vs.* 1.30% per 100 patient years in control arm HR 0.37 95% CI 0.14 to 0.96 *p* = 0.03	Device closure is not superior to medical therapy (intention‐to treat‐analysis) Device closure is superior to medical therapy (as‐treated analysis)
CLOSE (2017) [34] n = 663	Any approved PFO device *vs.* Antiplatelets: aspirin or Clopidogrel or aspirin with dipyridamole-ER *vs.* Anti-coagulation: Warfarin* or DOACs	5.3 years	Recurrent fatal or non-fatal stroke	0 recurrent strokes in device arm 6.2% recurrent strokes in antiplatelet arm HR 0.03 95% CI 0.00 to 0.26 *p* < 0.001	Device closure is superior to medical therapy.
REDUCE (2017) [35] n = 664	Helex Septal Occluder and Cardioform Septal Occluder *vs.* Aspirin or Clopidogrel or Aspirin with Dipyridamole	3.2 years^£^ (2–5 years)	Recurrent clinical strokes or NBI (Clinical + silent infarcts)	Recurrent Stroke: 1.4% in device arm *vs.* 5.4% in control arm HR 0.23 95% CI 0.09 to 0.62 *p* = 0.002 NBI: 5.7% in device arm *vs.* 11.3% in control arm RR 0.44 95% CI 0.24 to 0.81 *p* = 0.002	Device closure is superior to antiplatelet therapy
DEFENSE PFO (2018) [36] n = 120	Amplatzer PFO Occluder *vs.* Aspirin or aspirin and Clopidogrel, or aspirin and Cilostazol, or Warfarin*	2.8 years^£^ (0.9-2.1 years)	Composite of stroke, vascular death or TIMI-major bleeding during 2 year follow-up	2-Year event rate: 0% in device arm 12.9% in control arm *p* = 0.013	Device closure is superior to medical therapy

**Note:** A brief overview of randomized trials comparing percutaneous PFO closure in cryptogenic stroke. CI – Confidence interval; CLOSE – Closure of Patent Foramen Ovale or Anticoagulants *Versus* Antiplatelet Therapy to Prevent Stroke Recurrence; CLOSURE I – Evaluation of the STARFlex Septal Closure System in Patients with a Stroke and/or Transient Ischemic Attack Due to Presumed Paradoxical Embolism through a Patent Foramen Ovale; DEFENSE – PFO, Device Closure *Versus* Medical Therapy for Cryptogenic Stroke Patients With High‐Risk Patent Foramen Ovale; DOAC – Direct oral anticoagulants; ER – Extended release;

HR – Hazard ratio; n – Number of patients; NBI – New brain infarcts; PC – Percutaneous Closure of Patent Foramen Ovale Using the AMPLATZER PFO Occluder with Medical Treatment in Patients with Cryptogenic Embolism; PFO – Patent foramen ovale; REDUCE – GORE HELEX Septal Occluder/GORE CARDIOFORM Septal Occluder and Antiplatelet Medical Management for Reduction of Recurrent Stroke or Imaging‐Confirmed TIA in Patients With Patent Foramen Ovale (PFO); RESPECT – Randomized Evaluation of Recurrent Stroke Comparing PFO Closure to Established Current Standard of Care Treatment; RR – Relative risk; TIA – Transient ischemic attack; TIMI – Thrombolysis in myocardial infarction.

* – target international normalized ratio 2-3.

^£^ – Median follow-up reported.

**Table 2:** Overview of 5-Year Follow-up from RESPECT and REDUCE Trials

**Study, Year and Size**	**Closure Device**	**Median Follow-up**	**Primary Endpoint**	**Results**	**Conclusion**
RESPECT (Long term follow-up - 2017) [37] n = 980	Amplatzer PFO Occluder *vs*. Aspirin, clopidogrel, warfarin (target international normalized ratio 2-3) or aspirin plus dipyridamole	5.9 years	Composite of recurrent nonfatal ischemic stroke, fatal ischemic stroke, or early death after randomization	Intention to treat Event rate 0.58% in device arm *vs.* 1.07% in control arm per 100 patient years HR 0.55 95% CI 0.31 to 0.99 *p* = 0.046 Recurrent ischemic stroke of unknown mechanism Event rate 0.32% in device arm *vs.* 0.86% in control arm per 100 patient years HR 0.38 95% CI 0.18 to 0.79 *p* = 0.007	Device closure is superior to medical therapy to prevent recurrent ischemic strokes on long term follow-up
REDUCE (2021) [38] n = 664	Helex Septal Occluder and Cardioform Septal Occluder vs. Aspirin or Clopidogrel or Aspirin with Dipyridamole	5 years	Recurrent clinical strokes	1.8% in device arm 5.4% in control arm HR 0.31 95% CI 0.13 to 0.76 *p* = 0.007	Device closure is superior to antiplatelet therapy NNT with device to prevent one recurrent stroke is 25

Table **2**: Long-term follow-up of RESPECT and REDUCE trials. CI – Confidence interval; HR – Hazard ratio; n – Number of patients; NNT – Number needed to treat; PFO – Patent foramen ovale; REDUCE – GORE HELEX Septal Occluder/GORE CARDIOFORM Septal Occluder and Antiplatelet Medical Management for Reduction of Recurrent Stroke or Imaging‐Confirmed TIA in Patients With Patent Foramen Ovale (PFO); RESPECT – Randomized Evaluation of Recurrent Stroke Comparing PFO Closure to Established Current Standard of Care Treatment.

**Table 3:** Variables in calculating RoPE score.

**RoPE SCORE**	
**Patient Characteristics**	**Points**
No history of stroke or transient ischemic attack	1
No history of hypertension	1
No history of diabetes	1
Cortical infarct on imaging	1
Nonsmoker	1
Age (years)	-
18-29	5
30-39	4
40-49	3
50-59	2
60-69	1
≥70	0

**Note:** Variables involved in calculation of RoPE score.

RoPE – Risk of paradoxical embolism.

Maximum score = 10, Minimum score = 0

From: Kent DM, Ruthazer R, Weimar C, Mas JL, Serena J, Homma S, Di Angelantonio E, Di Tullio MR, Lutz JS, Elkind MS, Griffith J, Jaigobin C, Mattle HP, Michel P, Mono ML, Nedeltchev K, Papetti F, Thaler DE. An index to identify stroke-related *vs.* incidental patent foramen ovale in cryptogenic stroke. Neurology. 2013 Aug 13;81(7):619-25. doi: 10.1212/wnl.0b013e3182a08d59. Epub 2013 Jul 17. PMID: 23864310; PMCID: PMC3775694.

## LIST OF ABBREVIATIONS

ASD: Atrial Septal Defect
TTE: Transthoracic Echocardiogram
TEE: Transesophageal Echocardiogram
US: United States
